# Double trouble - management of perinephric hematoma and renal vein thrombosis post percutaneous renal biopsy

**DOI:** 10.1186/s12882-022-02935-z

**Published:** 2022-09-09

**Authors:** Muhammad Imran Kamarudin, Chandran Nadarajan, Mohamed Ashraf Mohamed Daud

**Affiliations:** 1 School of Medical Sciences, Hospital Universiti Sains Malaysia, Kubang Kerian, Kota Bharu, Kelantan Malaysia; 2 Department of Internal Medicine, Hospital Universiti Sains Malaysia, Kubang Kerian, Kota Bharu, Kelantan Malaysia; 3 Department of Radiology, Hospital Universiti Sains Malaysia, Kubang Kerian, Kota Bharu, Kelantan Malaysia; 4 Department of Surgery, Urology Unit, Hospital Universiti Sains Malaysia, Kubang Kerian, Kota Bharu, Kelantan Malaysia

**Keywords:** Percutaneous renal biopsy, Perinephric hematoma, Renal vein thrombosis, Lupus nephritis, Case report

## Abstract

**Background:**

Performing percutaneous renal biopsy procedures in lupus nephritis (LN) and nephrotic syndrome presents a unique challenge to the nephrologist because of the risk of bleeding from the procedure and the hypercoagulable state in hypoalbuminemia. The management of a patient with venous thrombosis with perinephric hematoma post renal biopsy can be difficult if occurred.

**Case presentation:**

We are presenting a case of perinephric hematoma following percutaneous renal biopsy in a 23-year-old man with lupus nephritis, nephrotic syndrome, and lower limbs deep vein thrombosis (DVT). The patient developed persistent frank haematuria, flank pain and acute urinary retention post-procedure. We have withheld his oral warfarin three days before the procedure, and no anticoagulation was given subsequently. Initial CT Angiography (CTA) renal showing stable hematoma and no visible evidence of vascular injury. Three weeks later, the patient still has persistent frank haematuria and a repeated CTA renal revealed new bilateral renal vein thrombosis. Considering the high risk of worsening symptomatic venous thrombosis, we gave subcutaneous enoxaparin sodium and restart oral warfarin despite ongoing haematuria. The frank haematuria resolved within two days of anticoagulation with no radiological evidence of worsening of the perinephric hematoma. The follow-up ultrasonography a month later showed resolution of the hematoma and renal vein thrombosis with no adverse effect.

**Conclusion:**

Our experience, in this case, highlighted the importance of case selection for percutaneous renal biopsy among high-risk patients. Additionally, a prolonged frank haematuria in post-renal biopsy with nephrotic syndrome warranted a reassessment, as a clinical presentation of post-procedure perinephric hematoma and renal vein thrombosis can overlap. We also demonstrated that restarting anticoagulation earlier than four weeks in a patient with renal vein thrombosis and post-renal biopsy perinephric hematoma can be safe in the selective case.

## Background

The overall risk of complications with percutaneous renal biopsy is relatively low, and the prevalence for severe complications in literature is usually 1-1.5% only [1–3]. Perinephric hematoma is one of the complications that can occur post renal biopsy. While in most cases it is self-limiting, severe sequelae from perinephric hematoma can cause severe renal injury and nephrectomy. There are possible factors that implicated a higher risk for hematomas, such as high BMI, abnormal urea, and renal size [4].

The role of renal biopsy in lupus nephritis (LN) is essential for disease class recognition, treatment options, and prognostic projection. Despite the overall benefit of histology diagnosis in LN, the physician must weigh the benefit versus the risk for percutaneous renal biopsy in high-risk patients such as LN with significant nephrotic syndrome or on anticoagulation.

Renal vein thrombosis is not an uncommon complication in nephrotic syndrome patients because of the pro-thrombotic state that accompanies hypoalbuminemia [5]. Renal vein thrombosis can manifest in a range of presentations, from haematuria to acute renal failure with severe flank pain. The concurrent presence of renal vein thrombosis and post-renal biopsy perinephric hematoma can become very challenging to manage as the treatment for both conditions can be contradictory and may worsen one another.

## Case presentation

A 23-year-old man with two years history of primarily joint and cutaneous involvement of Systemic Lupus Erythematosus (SLE), presented with a one-month history of pedal oedema and frothy urine. 24-hour urine protein revealed 3.34g of protein in 2500ml urine. His biomarkers were; sodium 138mmol/L, potassium 4.5mmol/L, urea of 3.3mmol/L, creatinine 72umol/L, serum albumin of 34g/L and total cholesterol of 8.4 mmol/L. The connective tissue markers result was ANA positive at titre 1:320, anti-dsDNA 87.69IU/ml, C3 0.76g/L and C4 0.4g/L. He was diagnosed with active LN with nephrotic syndrome. We started him on oral prednisolone 1mg/kg and the percutaneous renal biopsy was done two weeks later by the intervention radiology team. The biopsy was uneventful, but the patient developed left leg deep vein thrombosis (DVT) one day after the procedure. The patient was clinically still nephrotic, with serum albumin of 26g/L, and 24-hour urine protein was 6.93g/L. The screening tests for antiphospholipid antibodies were negative. He was started on oral warfarin two days after the biopsy.

The renal biopsy sample, however, was suboptimal and contained only one glomerular, thus not suitable for interpretation. Based on his clinical pictures, we treated him as LN class III or IV and started him on oral Mycophenolate mofetil induction of 1500mg twice daily and continue oral prednisolone with a tempering dose. Despite 12 months of Mycophenolate mofetil, the patient has persistent heavy proteinuria and recurrent symptomatic nephrotic syndrome. The patient later developed a severe skin infection while on oral Mycophenolate mofetil, requiring cessation of the medication. He also suffered from steroid toxicity with significant facial swelling, body striate and weight gain while on 6 months of multiple courses of high-dose oral prednisolone. In addition. repeated bedside Doppler ultrasonography study shows persistent left leg DVT. Because of failing to achieve disease remission with 24-hour urine protein worsening to 12.61g/L, we reattempt the renal biopsy. Since the patient already withheld oral warfarin three days before the biopsy, bridging therapy of IV unfractionated heparin was initiated. We stopped IV unfractionated heparin 4 hours before the biopsy. The blood investigation prior to the procedure was recorded as urea 7.7mmol/L, creatinine 95umol/L, Albumin 22g/L, Hb 11.9g/dl, Platelet 158x10^9/l, PT 11.40, INR 0.84, and APTT 28.60.

The intervention radiology team performed the biopsy using a semiautomatic biopsy needle size 18G. Four passes cultivating two good 1cm core and two fragmented tissues were made to the right renal under ultrasound guidance. In the immediate post-procedure period, ultrasonography detected the hematoma at the right upper pole perinephric region and along the needle track which measures 3.4cm x 5.1cm. The hematoma was not expanding in the repeated scan done half an hour later. Clinically, the patient had minimal discomfort, his blood pressure and his heart rate were 135/75mmHg and 94bpm. He was sent to the ward for close observation and immediately developed frank haematuria on the first voiding post-procedure. An urgent computed tomography angiography (CTA) renal showed no evidence of active arterial bleeding, pseudoaneurysm, fistula or pooling of contrast from the right renal vessel. The repeated coagulation profile also was normal. Repeated antiphospholipid antibodies screening was also negative. Despite the CTA finding, the patient’s frank haematuria worsened, and he required bladder irrigation because of acute urinary retention from persistent blood clots in the urine. In response to these events, we did not restart any anticoagulation therapy post-procedure.

The patient’s frank haematuria and dull flank pain are persistent after three weeks post-event. Despite ongoing haematuria, the patient’s haemoglobin level remained within normal ranged, and he was hemodynamically stable. His creatinine level has been normal and stable throughout the event despite multiple exposures to contrast. Repeated ultrasonography of the kidney showed persistent non-expanding perinephric hematoma. We arranged another CTA renal, and the scan showed a stable right perinephric hematoma with no evidence of ongoing bleeding or vessel injury, but with a new bilateral renal veins’ thrombosis seen. Given the new finding, we decided to re-anticoagulated the patient despite the ongoing presence of hematoma and haematuria. We started him on 5mg oral warfarin with subcutaneous enoxaparin sodium (1mg/kg twice daily) as bridging therapy. Within two days after restarting anticoagulation therapy, the frank haematuria has resolved completely. Repeated ultrasonography a month later showed resolution of perinephric hematoma, renal vein thrombosis and left leg DVT. Histopathological analysis of biopsy tissue was consistent with LN class IV (Diffuse proliferative nephritis with Activity Index 10, Chronicity Index 3). He was started on IV Cyclophosphamide Euro-lupus and achieved partial remission with current treatments.

## Discussion and conclusions

The dual complications post renal biopsy in our patient presented an interesting learning opportunity. One of the common clinical presentations for perinephric hematomas post-biopsy is the presence of haematuria which can also present in the case of renal vein thrombosis. The overlapping clinical presentation between perinephric hematoma and renal vein thrombosis can lead to a delay in making diagnoses and initiating suitable treatment, especially if renal vein thrombosis developed a little later. Our patient has prolonged frank haematuria and ipsilateral flank pain despite the radiological evidence showing stable hematoma and no visible vascular injury, thus leading to the need to find other causes for persistent frank haematuria. We need to emphasize that our patient has a significant risk for developing further thromboembolic events, as he has ongoing DVT, with persistent nephrotic syndrome and was on prolonged bed rest because of the ongoing bladder irrigation. Acute thrombosis in SLE with antiphospholipid syndrome is possible but negative antiphospholipid antibodies screening make it unlikely.

The next dilemma is regarding the best timing to start the anticoagulation therapy in a high-risk patient with iatrogenic perinephric haematoma and multiple venous thromboses. The consensus among the medical fraternity on the timing to restart anticoagulation regiment in post-procedural perinephric hematomas is very limited, but normally it is acceptable to wait up to six weeks before restarting anticoagulation again. There were cases of spontaneous perinephric bleeding in patients on anticoagulation reported in the literature, and for this reason, we were cautious on the timing to restart anticoagulation therapy in our patient [6–9]. In extra-renal hematoma such as intracranial hematoma, The American Heart Association and American Stroke Association suggested waiting at least four weeks before starting, albeit the evidence was only Class IIB [10]. Thus, most physicians will agree that four to six weeks would be the most acceptable time to restart anticoagulation in a patient with coexisting perinephric hematoma. However, in our patient, we believed his perinephric hematoma has stabilized and the ongoing haematuria is likely from the renal vein thrombosis. Thus, we have restarted anticoagulation therapy earlier, at three weeks post renal biopsy procedure to prevent further thromboembolic events.

In conclusion, we acknowledged that high-risk patients need special consideration before the percutaneous renal biopsy. Other methods of obtaining renal tissue such as a transjugular biopsy should be considered if necessary. Furthermore, among nephrotic patients, prolonged frank haematuria in radiologically stable hematoma with no vessel injury warranted differential diagnosis, such as renal vein thrombosis. We also have demonstrated that restarting anticoagulation slightly earlier than four weeks in a patient with renal vein thrombosis and post-renal biopsy perinephric hematoma can be safe in the selective case.

## Data Availability

Not applicable. All data generated or analysed during this study are included in this published article.

## Abbreviations

SLE: Systemic lupus erythematous
APLS: Antiphospholipid syndrome
DVT: Deep vein thrombosis
CTA: Computed tomography angiography
CBD: Continuous bladder drainage
